# DNA Methylation Profiles and Their Diagnostic Utility in BC

**DOI:** 10.1155/2019/6328503

**Published:** 2019-05-06

**Authors:** Ming Shan, Lei Zhang, Yang Liu, Chunyang Gao, Wenli Kang, Weiwei Yang, Yan He, Guoqiang Zhang

**Affiliations:** ^1^Department of BC Surgery, Harbin Medical University Cancer Hospital, Harbin, China; ^2^Department of Pathology, Harbin Medical University, Harbin, China; ^3^Department of Oncology, General Hospital of HeiLongjiang Province Land Reclamation Headquarter, Harbin, China

## Abstract

Biomarkers, including DNA methylation, have shown a great potential for use in personalized medicine for BC and especially for the diagnosis of BC in developing countries. According to the bisulfite sequencing PCR in twelve specimens (BC and matched normal tissues), nine genetic probes were designed to detect the frequency of methylation of the promoters in a total of 302 paired cases of BC and matched normal breast tissues. Finally, a total of 900 serum samples were used to validate the use of these methylation biomarkers for clinical diagnosis of BC. A high frequency of promoter methylation of *SFN*, *HOXA11*, *P16*, *RARβ*, *PCDHGB7*, *hMLH1*, *WNT5a*, *HOXD13*, and *RASSF1a* was observed in BC tissues. The methylation frequencies of *HOXD13* and *hMLH1* increased with the progression of BC. The methylation frequencies of *HOXD13* and *WNT5a* were significantly higher in BC. We found that methylation modification-positive samples were most consistently associated with luminal BC. Finally, we confirmed that *RASSF1a*, *P16*, and *PCDHGB7* displayed a significant sensitivity and specificity as diagnostic biomarkers for BC (*P* < 0.001), and a panel that combined these three genes displayed increased significance (AUC, 0.781; *P* < 0.001). These data suggest that epigenetic markers in serum can potentially be used to diagnose BC. The identification of additional BC-specific methylated genes would improve the sensitivity and specificity of this approach. This study could also indicate that different molecular subtypes of BC are caused by distinct genetic and epigenetic mechanisms.

## 1. Introduction

Breast cancer (BC) is a complex and heterogeneous disease and a leading cause of death among women. Some regional surveys have indicated that the incidence of BC is also rising in Chinese women [1]. Approximately, the incidence of BC was 26.86% and the mortality of BC was 6.95% among Chinese women in 2015. High incidence is concentrated between 45 and 59 years old [2].

Carcinogenesis is a multistep process that results from the accumulation of genetic and epigenetic alterations [3]. Recent reviews have emphasized that epigenetic abnormalities might play an influential role in the earliest steps of cancer initiation and the progression of malignancies [4, 5], especially because the methylation of a normal allele can serve as a “second hit” that leads to gene inactivation when paired with mutations in the opposite allele [6]. Approximately 40-50% of human genes have CpG islands (CGIs) located in or near the promoter and/or first exon, and the methylation of these CGIs is critical to regulate the expression of these genes [7]. Alterations in the methylation status of DNA are among the most frequent molecular changes that are associated with human cancers [8].

In BC, many studies have investigated methylation patterns as potential biomarkers for detection, subtype classification, risk stratification, monitoring prognoses, and predicting susceptibility or responsiveness to a particular therapy [9, 10]. However, in spite of the promise of such biomarkers, several barriers continue to prevent rapid progress toward using these markers in clinical applications. Major limitations to the further development of these markers in clinical applications might be that many studies have focused on investigating the methylation patterns in circulating free DNA (cfDNA) derived from the serum of healthy women and women with BC, but studies rarely use benign breast tissues as the control to identify the potential clinical applications of using serum DNA methylation as a biomarker. In addition, these studies have investigated fewer BC and matched control specimens, and validation with larger patient cohorts has not been pursued [11, 12]. Other limitations include the utilization of different technologies by different laboratories, resulting in a range of detection sensitivities, a varying emphasis on quantitation, and the utilization of different sample processing methodologies and different reference materials as controls during analysis of hypermethylation degrees when using the same technology [13]. We therefore investigated a new diagnostic tool for BC and sought to overcome these barriers by using methylation genes as cancer biomarkers. We determined whether these genes could also be useful markers for predicting a prognosis in BC patients according to the progression of the cancer. These were *ductal carcinoma in situ* (DCIS), *invasive ductal carcinoma* (IDC), and *invasive ductal carcinoma plus lymph metastasis* (IDC-L).

In the present study, twelve candidate markers for BC (*SFN* (*14-3-3σ*), *HOXA11*, ARID1a, CBX7, DLC1, *P16*, *RARβ*, *PCDHGB7*, *hMLH1*, *WNT5a*, *HOXD13*, and *RASSF1a*) were studied with regard to their detection in BC tissues and matched serum samples. These genes have previously been shown to undergo cancer-specific methylation in breast tissues in the TCGA database [14] and other reports of clinical or fundamental studies [15–20]. These markers are representatives of a variety of cellular pathways, including DNA binding, cell cycle/checkpoint control, developmental regulation, chromatin binding, cell adherence, and cytokine activity. In addition, *HOXA11*, *HOXD13*, and *PCDHGB7* were confirmed as early methylated genes when human mammary epithelial cells (HMEC) converted into cancer cells in our previous study and in other studies [19]. We examined the methylation status of the promoters of these candidate genes in two independent sets (test and validation) using a total of 302 paired tissue/normal samples. A matched serum detection assay of the validation set (*n* = 194) was then used to confirm the results obtained for the top hypermethylated genes from both the test and the validation sets, to show the reliable cfDNA methylation markers that diagnose BC. Finally, we identified the methylation biomarkers that best differentiated BC in a total of 900 serum samples that included samples from 300 BC patients, 300 patients with benign breast diseases, and 300 healthy women.

## 2. Materials and Methods

### 2.1. Patients, Sample Collection, and DNA Extraction

All individuals signed surgical or clinical research consent forms allowing tissue and serum collection in accordance with the regulations approved by the IRB Committee of Harbin Medical University. This research was completed in compliance with the Helsinki Declaration. A brief outline of the study process is shown in Supplemental Figure 1. The study extended over biomarker development phases 1 and 2, which were based on Early Detection Research Network (ERDN) guidelines [13, 21]. All the tissue and serum samples were obtained from patients and healthy persons undergoing physical examination at the Affiliated Tumor Hospital of Harbin Medical University, Harbin, China, from 2014 to 2017. Fresh-frozen specimens derived from cancerous and self-pair normal breast tissues (≥5 cm distant from the tumor tissue) were obtained from patients who underwent a mastectomy for BC. The benign breast diseases included fibroadenoma, benign phyllodes tumors, mastopathy, papilloma, duct ectasia, and hamartoma (Supplemental Table 3). Healthy serum samples were acquired from the Affiliated Tumor Prevention and Treatment Institution of Harbin Medical University. All H&E slides were reviewed by two independent pathologists to determine the integrity of the tumor specimen (tumor content of >70%) and the normal tissue blocks, in which no tumor cells were observed.

All samples were classified as one of four types of primary BC lesions: (1) pure DCIS, 100 cases; (2) IDC, 100 cases; and (3) IDC-L, 102 cases. Genomic DNA was isolated from fresh-frozen primary breast tumors and matched normal breast tissues. Samples were pretreated with proteinase K (20 mg/mL) at 55°C overnight and DNA was then extracted using an AxyPrep™ Multisource Genomic DNA Miniprep Kit (Axygen Scientific Inc., CA, USA). Approximately 5 mL of peripheral blood was drawn into a blood collection tube prior to a physical examination or surgery, and all samples were transferred to the study laboratory within 4 hours of collection for processing. Circulating free DNA (cfDNA) was obtained from 1 mL of serum using a QIAamp Circulating Nucleic Acid Kit [22] (Qiagen, Hilden, Germany) according to the manufacturer's instructions.

### 2.2. Immunohistochemistry and Molecular Subtypes

The monoclonal ER antibody was obtained from Ventana (catalog no. 760-2596). The monoclonal PR antibody was obtained from Dako (catalog no. M3569). Nuclear labeling for ER positivity or PR positivity was required in greater than 1% of cells [23]. HER-2 IHC was performed using the Dako HercepTest kit according to the manufacturer's protocol. Cases were scored using the established criteria as 0, 1+, 2+, or 3+. Fluorescence in situ hybridization analysis to determine Her-2 amplification was performed on all 2+ (equivocal) cases using the PathVysion kit (Des Plaines, IL). To qualify as Her-2 positive in this study, a case had to demonstrate either a 3+ IHC score or a Her-2 fluorescence in situ hybridization amplification ratio of greater than 2.2. Cases were categorized into one of four categories based upon accepted and previously validated IHC surrogate profiles of BC. Luminal A tumors were immunoreactive for ER and/or PR and negative for Her-2 or low proliferation. Tissue that was ER+ and/or PR+, either Her2+ and/or highly proliferative, was considered luminal B tumors. The Her-2 subtype was defined as ER-, PR-, and Her2+. Basal-like tumor was the most controversial type. On the basis of the published criteria, basal-like cases were defined as tissues with a triple-negative phenotype (ER-/PR-/Her2-). We therefore used triple-negative BC (TNBC) instead.

IHC for p53 (Ventana, monoclonal antibody, catalog no. 760-2542) and Ki-67 (Ventana, monoclonal antibody, catalog no. M7240) only showed nuclear labeling. For p53, a labeling score indicating that >30% of the nuclei were labeled was defined as aberrant overexpression (which correlates well but not perfectly with the presence of p53 mutation) [24]. The Ki67 cut-off point was 20%, and this was used to designate a tumor as highly proliferative when assigning samples to subtype groups [25].

### 2.3. Bisulfite Treatment, Sequencing, and MethyLight

Bisulfite conversion of genomic DNA was performed using an EZ DNA Methylation kit (Zymo Research, Orange, CA, USA) according to the manufacturer's instructions. Converted DNA was amplified using PCR as described in Supplemental Excel 1. For each BSP, ten positive clones were sequenced in both directions by the Life Technologies Lab (Invitrogen, Burlington, ON, CA).

According to the results of BSP sequencing, we selected the probable promoter CpG islands that contained the methylated variant sites to design probes for each gene (Supplemental Figure 1). A detailed list of the nucleotide sequences corresponding to the MethyLight primers and probes in the promoter or 5′ end region of all analyzed loci is provided in Supplemental Excel 2. TaqMan MGB (Applied Biosystems, Foster City, CA, USA) PCR was performed using primers specific for the bisulfite-converted methylated sequence of a particular locus. *Globin* reference primers were used separately. The TaqMan MGB probes showed a significant improvement in assay specificity, and their smaller size allowed for a more flexible assay design.

MethyLight is highly specific, sensitive, and reproducible. It can also rapidly detect biologically relevant information in patient samples. MethyLight is a PCR-based method that requires only very small amounts of DNA of modest quality, and this makes it compatible with small biopsies and paraffin-embedded tissues [26]. MethyLight could therefore be a utility tool for use in clinical applications [13]. The majority of studies that have used percentage of methylated reference (PMR) as a method for evaluating methylation have reported positive results. But the cut-off value for PMR varies when used with MethyLight in different studies [15, 27–29]. This is likely the result of not using self-matched normal tissue as a control in studies that instead use *SssI*-treated human peripheral white blood cell DNA from the same person or from healthy people as the control. This comparison may not accurately reflect positive methylation cases, because methylation modification is influenced by many factors, including lifestyle, environmental exposure, ethnicity, age, and tissue heterogeneity [26, 30]. In this study, we compared BC tissue to matched normal breast tissue (distant from tumor mass ≥ 5 cm) from the same person, and the percentage of samples that were methylated at a specific locus was statistically calculated using the 2^-ΔΔCt^ method, where ΔΔCt = (CT_Target gene_ − CT_Reference_) sample − (CT_Target gene_ − CT_Reference_) control (matched normal tissue from the same patient) [31]. All samples were assayed in duplicate, and to validate the results of the 2^-ΔΔCt^ method, the amplification efficiencies of the test genes and a reference gene, Globin, were examined using serial dilutions of DNA over a 100-fold range and using gene-specific primers for each gene and Globin. The ΔCt (CT_Target gene_ − CT_Reference_) was calculated for each DNA dilution, and a plot of the log DNA dilution vs. ΔCt was constructed. A cut-off value of ≥1.5 [32, 33] (allelic gene methylation) was determined to indicate a positive result. The analysis of cfDNA methylation frequency was also performed using the MethyLight method. We used 2^-ΔCt^ (ΔCt was calculated as CT_Target gene_ − CT_Reference_) in a ROC curve analysis to determine both sensitivity and specificity in comparison of results between BC and control samples (including healthy women and patients with benign breast diseases).

### 2.4. Statistical Analysis

Data were analyzed using Student's *t*-tests, Fisher's exact tests, Kruskal-Wallis *H*, ROC curve analyses, and Mann-Whitney *U* tests. All tests were performed using SPSS 17.0. A *P* value of <0.05 was considered significant.

## 3. Results

### 3.1. Prescreening of the Promoter CpG Islands of Candidate Genes to Select Methylation Targets Using BSP

From the TCGA database, gene methylation biomarkers identified for the diagnosis of other tumors in the previous studies, and the distinct methylation genes identified during the conversion from human normal mammary epithelial cells to BC cells, we selected *SFN* (14-3-3*σ*), *HOXA11*, ARID1a, CBX7, DLC1, *P16*, *RARβ*, *PCDHGB7*, *hMLH1*, *WNT5a*, *HOXD13*, and *RASSF1a*, which represent a variety of different pathways that are involved in cancer (Supplemental Excel 3). Initially, we evaluated the CpG islands of all of these genes [34], and the highly dense regions containing the CpG sites in the CpG islands were sequenced using BSP in six paired cases of BC tissues and matched normal breast tissues (Supplemental Figure 2). DLC1 was eliminated because the highly dense CpG region could not be amplified using BSP. We then selected variant methylation sites that were methylated in at least half of the BC tissues and unmethylated in the matched breast normal tissues to design the probes for MethyLight (Supplemental Table 1).

### 3.2. Determination of Methylation Frequency in an Appropriate Gene Evaluation Set of Patients

Next, all residual tissue specimens were divided into two data sets: the test set (108 paired cases of BC tissues and matched normal breast tissues) and the validation set (194 paired cancer tissues and matched normal tissues). All of these samples were obtained from BC patients aged 40-60 years old to rule out the effect of age on DNA methylation. The other clinicopathological factors, including the pathological type, histological grade, BMI, and tumor size, were not different (*P* > 0.05, Supplemental Table 2) between the test set and the validation set.

We investigated the methylation frequency of eleven genes between BC tissues and matched normal breast tissues in the test set using MethyLight. Significantly high methylation frequencies were detected for nine genes in BC tissues from the test set. Moreover, we next confirmed these results using the same nine methylation probes in the validation set, and we found that they also displayed the high methylation frequencies (Supplemental Table 4). In conclusion, a total of nine methylated genes, including *SFN*, *HOXA11*, *P16*, *RARβ*, *PCDHGB7*, *hMLH1*, *WNT5a*, *HOXD13*, and *RASSF1a*, were methylated with significantly higher frequency in BC tissues than in matched normal breast tissues from 302 BC patients. The average methylation frequencies for all of the genes in the BC tissue group are shown in Supplemental Table 5. Among all of these markers, *PCDHGB7* was most often methylated (78.81%), whereas the lowest methylation frequency was observed for *WNT5a* (28.48%).

### 3.3. Methylation Frequency during the Progression of BC

**Figure 1:**
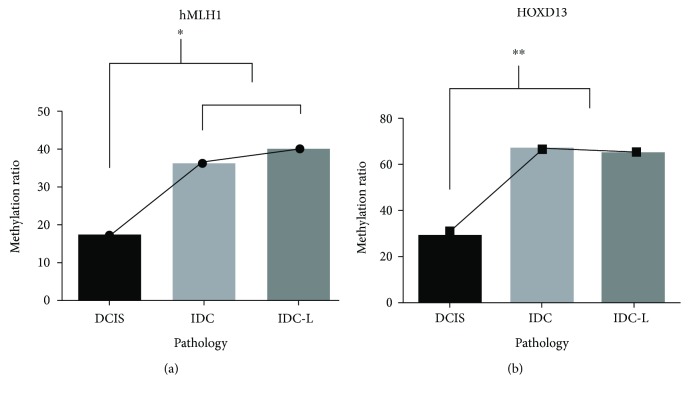


We categorized all of the malignant samples into four groups according to the histopathology of BC, including DCIS, IDC, and IDC-L. The results of the methylation frequency analysis for all nine genes is illustrated in Table 1. All of the genes displayed widespread aberrant promoter CpG island methylation. The frequency of *HOXD13* and *hMLH1* methylation significantly increased with the progression of the disease from in situ to invasive cancer (*P* < 0.001 and *P* < 0.05, Figure 1), but there was no significant difference between IDC and IDC-L.

### 3.4. Methylation Profiles Associated with Molecular Subtypes and Clinicopathological Features

**Figure 2:**
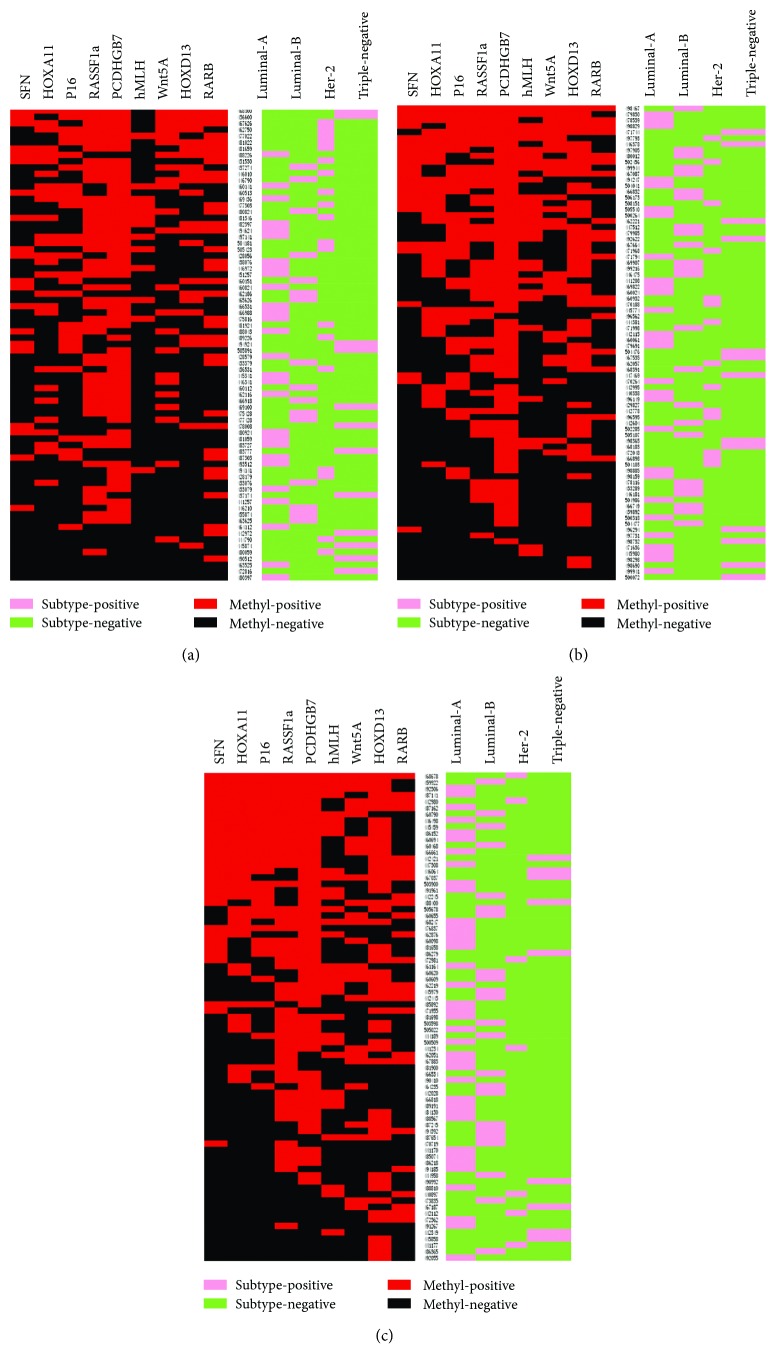


**Figure 3:**
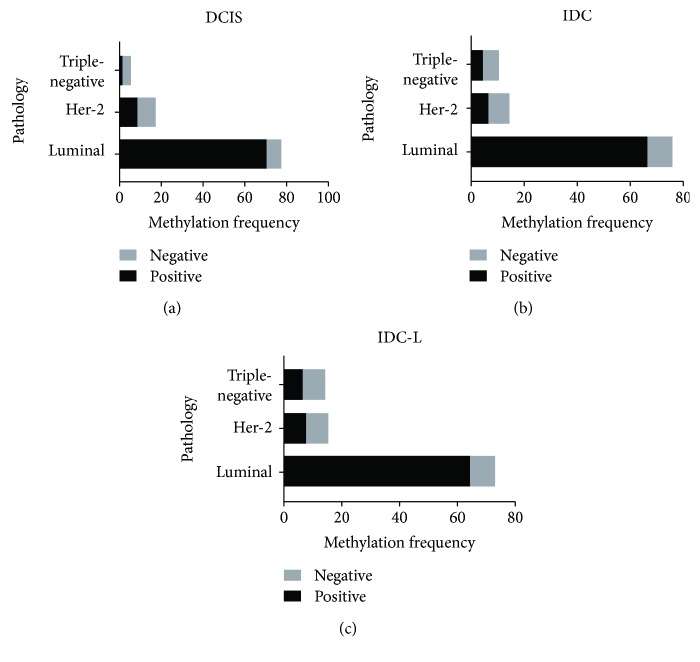


In each group (DCIS, IDC, and IDC-L), we classified three subgroups according to the coexistence of methylation between genes: coexistence of one to three methylation genes, four to six methylation genes, or seven to nine methylation genes (Supplemental Table 6). We found that the category with the fewest genes was the group indicating the coexistence of seven to nine methylated genes in the DCIS group (5%). To exclude contingency and on the basis of the prior studies [35], we determined that samples in which at least three genes were simultaneously methylated were likely to be affected by epigenetic modifications (especially DNA methylation modifications) and we named these “methylation modification-positive samples.” Next, we analyzed the specimens that clustered with the BC molecular subtypes in different groups (Figure 2). We found that methylation modification-positive samples were consistently the luminal type of BC (Figure 3) in the DCIS, IDC, and IDC-L groups.

### 3.5. Evaluation of the Consistency of Methylation Frequency between BC Tissues and Matched Serum and Determination of the Best-Performing Methylation Probes in BC Diagnoses

Based on the above data, we have shown that gene methylation frequencies are significantly higher in BC tissues. Next, we considered whether methylation can be used as a diagnostic marker of BC. We used specific probes to assess the methylation of nine genes in matched serum samples in the validation set using MethyLight. The majority of the genes that were methylated in the BC tissue were also methylated in the matched cfDNA obtained from serum (in the gene methylation-positive tissues). The same genes displayed higher average frequencies in the matched serum, including *PCDHGB7*, *P16*, and *RASSF1a* (Table 2). Meanwhile, the frequency of *HOXA11* or *WNT5a* methylation was low in serum, even though the frequency of methylation of these markers in the matched BC tissues was high. In addition, *HOXD13* was only methylated in 1 cfDNA sample, and *RARβ* was only methylated in 3 samples of cfDNA. The methylation frequency of *hMLH1* was 33.33% in serum and 35.57% in tissues. Finally, the observation that frequencies increased along with the progression of BC, as observed in tissues and illustrated in Figure 3, did not recur in the serum methylation study.

**Figure 4:**
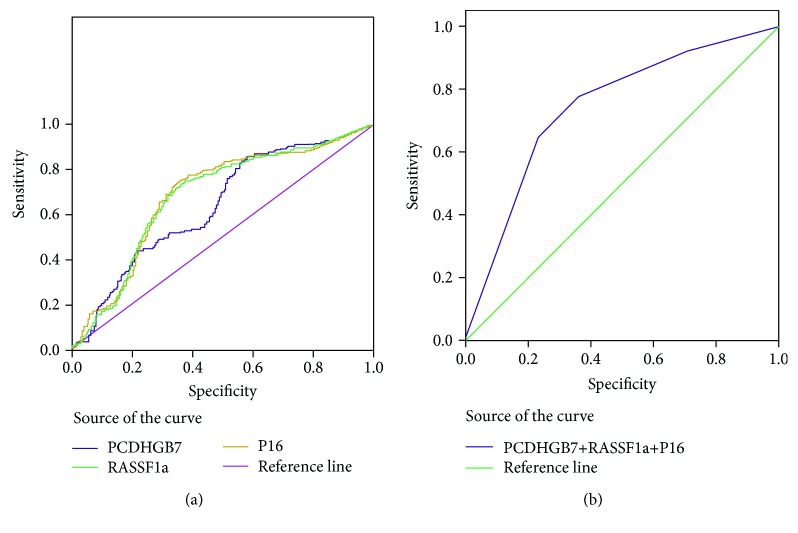


According to the frequent study of methylation genes in breast cancer tissues and serum samples, we selected genes that had a higher methylation frequency in both the breast cancer tissues and the matched serum samples to explore the clinical utility of using such methylation biomarkers to diagnose breast cancer. *PCDHGB7*, *P16*, and *RASSF1a* (although the methylation frequency of *RASSF1a* was lower in the serum in this study, it was generally high in breast cancer tissues) were selected. We used an expanded set of serum samples that included 300 breast cancer samples, 300 samples from age-matched healthy controls, and 300 samples from age-matched patients with benign breast diseases. In the ROC curves corresponding to the three analyzed genes (Figure 4(a)), *RASSF1a* showed a sensitivity of 75%, a specificity of 62.5%, and an area under the curve (AUC) of 0.682 (95% CI, 0.645 to 0.719, *P* < 0.001). The sensitivity and specificity of *P16* were 75% and 64.33%, respectively, and the AUC was 0.687 (95% CI, 0.650 to 0.724, *P* < 0.001). *PCDHGB7* showed the highest sensitivity (84.33%). This is in accordance with our results showing that this marker showed the highest methylation frequency in breast cancer tissues (Supplemental Table 5). However, the specificity of serum *PCDHGB7* was not very high (60.33%), and the AUC for this marker was 0.660 (95% CI, 0.630 to 0.678, *P* < 0.001). Next, we performed an ROC curve analysis for a three-gene panel to determine its sensitivity and specificity for diagnosing breast cancer (Figure 4(b)). According to this analysis, the three-gene panel discriminated between breast cancer patients and controls with a sensitivity of 82.67% and a specificity of 77.83% (AUC, 0.781; 95% CI, 0.757 to 0.796, *P* < 0.001). This combination of three different methylation markers maximized their significance in the clinical diagnosis of breast cancer.

## 4. Discussion

The recent report has indicated that the incidence of BC in developing countries is stably increasing [35]. However, distinctive features related to BC in Asian women relative to women of predominantly European ancestry include Asian women who have larger tumors and a more advanced tumor stage at diagnosis, and these characters are associated with delayed diagnosis or more aggressive disease [1, 22, 36]. Approximately 75% of cases in Asian women are diagnosed at late and untreatable stages (clinical stages III and IV) [37, 38]. This may be due to lack of awareness, limited healthcare infrastructure, inadequate manpower, and the uneven distribution of resources. Mammography has been the “gold standard” for BC detection for decades, and it is commonly applied in western countries. However, it is not suitable for use in developing countries as a diagnostic tool for BC because of limitations, including age, the density of breast tissues, socioeconomic factors, and medical resources [34, 39]. It is therefore necessary to explore efficacious, economical, convenient, and practical diagnostic methods that are suitable for use in developing countries.

In this study, we screened the methylation status of nine genes belonging to different molecular pathways in different pathological BC and matched normal tissues. This could raise the accuracy of such biomarkers for determining a diagnosis or prognosis in BC [40, 41]. Although *RASSF1a*, *RARβ*, *SFN*, *hMLH1*, and *P16* have been widely detected in different studies [18, 30, 42, 43] and in different people [27, 44, 45], we report important data regarding the frequency of the methylation of genes in BC. We found that *HOXA11*, *PCDHGB7*, and *HOXD13* are also highly methylated in BC tissues, which was rarely reported in the prior studies, especially in DCIS. Furthermore, we show that not all of the genes that are methylated in tumor tissues are also highly methylated in serum cfDNA, as the case for *HOXD13* and *HOXA11*. Although knowledge of the underlying mechanisms involved in determining the levels of these genes in circulating DNA is still limited [46], some evidence suggests that cfDNA is released from tumors as a glyconucleoprotein complex, which might protect it from degradation by nucleases [47]. It remains unclear whether the release of tumor DNA into serum is associated with tumor necrosis, apoptotic cell death, or other selective cellular processes. Because it is presumably shed from the original primary tumor, cfDNA might be fragmented and the quantity of cfDNA is greatly reduced. It has therefore been suggested that the clinical utility of using methylated biomarkers to diagnose BC must be confirmed in the serum and not just in BC tissues. For diagnostic biomarkers of BC, we selected *RASSF1a*, which is widely used as a methylation biomarker for diagnosing BC in western countries, and *P16* and *PCDHGB7* because they are highly methylated in both BC tissues and serum. *RASSF1a* and *P16* displayed a significant utility for diagnosing BC, as found in the prior studies. However, the sensitivity and specificity observed in this study were different from that found in the previous studies [16, 48, 49]. The most important reason for this discrepancy could be that we added benign controls in this study, and this may have reduced the sensitivity and specificity of these markers in contrast with results that compare only BC and healthy samples. Other reasons could be differences in the race of the sample population or their environment, difference in methodology, and differences in the targets being investigated. Hence, in future research aimed at investigating methylation to determine diagnostic biomarkers in BC, we strongly suggest that samples should contain matched samples from patients with benign diseases. The methylation frequency observed in *PCDHGB7* may be the first time that methylation has been detected in BC tissue and serum and found to be an effective methylation biomarker for the diagnosis of BC. In addition, we also investigated the mRNA expression of *PCDHGB7* in BC tissues to confirm its methylation in BC. The results showed that *PCDHGB7* was expressed at low levels in most BC tissues (approximately 80%, Supplemental Figure 3). Finally, we confirmed that a panel of methylated biomarkers that included these three genes showed the best sensitivity and specificity for the diagnosis of BC. Therefore, in future studies, we will add more effective methylation markers to increase the sensitivity and specificity of this panel for use in diagnosing BC.

Many studies have reported that the frequency of methylation should significantly increase in parallel with the progression of cancer [19, 33] and that methylation could therefore potentially be used as a predictor during the determination of a prognosis in BC. In our study, we showed that *HOXD13* and *hMLH1*, in BC tissue methylation detections, demonstrate this phenomenon. In particular, methylation of *HOXD13*, a member of the HOX family, significantly increased in parallel with the progression of BC (from in situ to metastasis). This example was also just reported by Gupta et al. [50] in *Nature*. However, this phenomenon did not reappear for either *HOXD13* or *hMLH1*in the analysis of methylation in serum. As we discussed above, this reminds us that accordance in methylation profiles between tissues and the matched serum samples is not perfect, as reported by Korshunova et al. [51]. Many methylation biomarkers that have been detected in BC tissues in the prior reports may not be suitable for clinical diagnoses of BC unless they are also analyzed in the serum. We also found that the methylation frequency of *RARβ* showed a significant variation: 43.45% in DCIS, 24.78% in IDC, and 34.68% in IDC-L, and these results followed the progression of BC. This may be the result of chance, or cyclic methylation modification mechanisms might be present, as reported in the prior studies [52, 53], in the *RARβ* promoter region, and these must be clarified in the future.

Finally, by performing an unsupervised clustering analysis of DCIS, IDC, and IDC-L, we found that luminal, Her-2, and triple-negative tumors had different methylation profiles. We synchronously clustered at least three methylation genes with different functions in a single specimen, relative to BC subtypes. The highest methylation frequencies were usually observed in luminal tumors. Her-2 and triple-negative BC samples displayed low methylation frequencies in general, and this result may be compatible with results indicating they have unstable and aberrant genomes, which may result from reduced transposon silencing. The association between methylated modification profiles and different subtypes has been mentioned in many previous investigations [40, 54, 55] because it could indicate that different molecular subtypes of BC could be caused by distinct genetic and epigenetic mechanisms [56].

## Figures and Tables

**Table 1:** Methylation frequencies for the nine genes in breast cancer patients.

Methylated gene	Breast cancer tissue (*302 cases*) methylation frequency (%)		
	DCIS	IDC	IDC_L
*SFN*	*25.67*	*27.35*	*36.67*
*HOXA11*	*38.23*	*40.67*	*45.34*
*P16*	*37.45*	*44.5*	*42.11*
*RASSF1a*	*64.45*	*54.38*	*68.45*
*PCDHGB7*	*75.56*	*83.45*	*76.45*
*hMLH1*	**23.55**	**40.57**	**47.43**
*Wnt5a*	**31.34**	**34.56**	**36.45**
*HOXD13*	**32.34**	**65.33**	**66.78**
*RARβ*	*43.45*	*24.78*	*34.68*

**Table 2:** Methylation frequencies of the same genes in the matched serum and methylation-positive BC tissues.

Methylated gene	Breast cancer tissue (validation test, 194 cases)		Methylation frequency (%)	Matched serum		Methylation frequency (%)
	Methyl case	Unmethyl case		Methyl case	Unmethyl case	
*SFN*	*61*	*133*	*31.44*	*35*	*26*	**57.38**
*HOXA11*	*88*	*106*	**45.36**	*15*	*73*	*17.01*
*P16*	*86*	*108*	**44.33**	*48*	*38*	**55.81**
*RASSF1a*	*117*	*77*	**60.31**	*43*	*74*	**36.75**
*PCDHGB7*	*156*	*38*	**80.41**	*79*	*77*	**50.64**
*hMLH1*	*69*	*125*	*35.57*	*23*	*46*	**33.33**
*Wnt5a*	*57*	*137*	*29.38*	*12*	*45*	*21.05*
*HOXD13*	*90*	*104*	**46.39**	*1*	*89*	*1.11*
*RARβ*	*71*	*123*	*36.6*	*3*	*68*	*4.23*

## Data Availability

(i) The following data (DATA TYPE) used to support the findings of this study are included within the article. The data are as follows: (1) methylation frequencies for the nine genes in sporadic and hereditary BC patients, (2) methylation frequencies of the same genes in the matched serum and methylation-positive BC tissues, (3) different methylation frequencies of HOXD13 and hMLH1 during the BC progression, (4) different methylation frequencies of HOXD13 and WNT5a between sporadic and hereditary BC, (5) DNA-methylated modification levels in different molecular subtypes of BC, and (6) ROC curve analysis of the three-gene methylation panel between BC, age-matched healthy and benign samples. (ii) The following data (DATA TYPE) used to support the findings of this study are included within the supplementary information file(s). The data are as follows: (1) the BSP PCR reaction primers and the system used to analyze the twelve genes., (2) MethyLight primers, probes, and the system used to analyze the twelve genes, (3) the signaling pathways of the candidate genes, (4) primers, probe sequences, and tested methylation sites for all genes, (5) clinicopathologic parameters of patients with BC in the test and validation sets, (6) methylated frequencies of nine genes in BC tissues from the test and validation sets, (7) methylation frequencies for the nine genes in BC patients, (8) the list of coexisting methylated genes in specimens with different histopathological types in sporadic and hereditary BC, (9) BC specimens in which at least three genes were simultaneously methylated in different subtypes (DCIS, IDC, IDC_L, and HpBC), (10) overview of the analyzed procedure, (11) the BSP analysis and the methylated sites chosen for all of the genes, (12) cluster analysis of methylated genes by BC subtypes in different groups ((a) DCIS, (b) IDC, (c) IDC-L, and (d) HpBC), and (13) differential expression of PCDHGB7 between BC tissues and matched normal breast tissues.

## Abbreviations

GWAS: Genome-wide association studies
CGI: CpG islands
cfDNA: Circulating free DNA
DCIS: Ductal carcinoma in situ
IDC: Invasive ductal carcinoma
IDC-L: Invasive ductal carcinoma plus lymph metastasis
HMEC: Human mammary epithelial cells
ERDN: Early Detection Research Network
TNBC: Triple-negative BC.

## Supplementary Materials

Supplementary Materials Supplemental Table 1: primers, probe sequences, and tested methylation sites for all genes. Supplemental Table 2: clinicopathologic parameters of patients with breast cancer in the test and validation sets. Supplement Table 3: clinical pathological characteristics of benign and normal control cases. Supplemental Table 4: methylated frequencies of nine genes in breast cancer tissues from the test and validation sets. Supplemental Table 5: methylation frequencies for the nine genes in breast cancer patients. Supplemental Table 6: the list of coexisting methylated genes in specimens with different histopathological types in breast cancer. Supplemental Figure 1: overview of the analyzed procedure. Supplemental Figure 2: the BSP analysis and the methylated sites chosen for all of the genes. Supplemental Figure 3: differential expression of PCDHGB7 between BC tissues and matched normal breast tissues.
