# Supplementary material for: DNA Methylation Profiles and Their Diagnostic Utility in BC
Source: Dis Markers. 2019 May 6;2019:6328503. doi: 10.1155/2019/6328503 (PMC6526564; doi:10.1155/2019/6328503)
Supplement: Supplementary Materials — Supplemental Table 1: primers, probe sequences, and tested methylation sites for all genes. Supplemental Table 2: clinicopathologic parameters of patients with breast cancer in the test and validation sets. Supplement Table 3: clinical pathological characteristics of benign and normal control cases. Supplemental Table 4: methylated frequencies of nine genes in breast cancer tissues from the test and validation sets. Supplemental Table 5: methylation frequencies for the nine genes in breast cancer patients. Supplemental Table 6: the list of coexisting methylated genes in specimens with different histopathological types in breast cancer. Supplemental Figure 1: overview of the analyzed procedure. Supplemental Figure 2: the BSP analysis and the methylated sites chosen for all of the genes. Supplemental Figure 3: differential expression of PCDHGB7 between BC tissues and matched normal breast tissues. [file 6328503.f1.doc]

**Supplemental Table 1. Primers, Probe Sequences and Tested Methylation Sites for All Genes**

| **GENE** | **Primer Forward** | **Primer Reverse** | **Probe Sequence** | **Methyl-site from TSS** |
| --- | --- | --- | --- | --- |
| **SFN** | *TATGAAAGGCGTCGTGGAGA* | *GATACTCACGCACCTCGAACC* | *AACGTGGTGGGCGGT* | *+ 221 bp* |
| **Wnt5a** | *GGTGTGAATGAATTGGGGGTAT* | *CTTTCCAACCCCAAATATAAACG* | *TTTGTTTCGGGTTATAGTTGA* | *- 178 bp* |
| **hMLH1** | *TGAGGYGGYGATAGATTAGG* | *TCCCCTTACRACCTTTCTAACR* | *AACGTTGGGTTTATTCGG* | *- 328 bp* |
| **HOXD13** | *GGGAATGGGAGGTGGATTTT* | *CCGCCGAAAACGTACCATT* | *TTGGGTCGGGAGTTAG* | *- 336 bp* |
| **RARβ** | *AGGGTTTGTATGTGTTTTTTTTGGA* | *AAAACGATATTCCGCCTACGC* | *AGATAGAAAGGCGTATAGAGG* | *- 475 bp* |
| **HOXA11** | *GTTTATGGGAGGGGGATTGC* | *TCAACTCTCGTCCCCACCTC* | *AGCGACGGATTTTTATT* | *- 480 bp* |
| **RASSF1a** | *TGAAGGTTRGAGATTTTTTGTTTTATT* | *GCAAAACACCAyGyGAAAATA* | *AGTAAATCGGATTAGGAGGG* | *- 194 bp* |
| **PCDHGB7** | *CGTTATTCGATTTCGGAGGAG* | *TTCACAAATAAATCCCCGCTCT* | *TGGTTAAGGGTTCGGTGGT* | *+ 123 bp* |
| **P16** | *GGGGTRGGGTAGAGGAGGT* | *AATCGACCTCCGACCGTAACT* | *TGTTGTTGGAGGCGGG* | *+ 360 bp* |
| **ARID1a** | *GGGGYGTTTTAGTYGTTTAG* | *GCRGAAAACCAAAATCAAAA* | *ATTGTTTGTTCGTTTGTTTGT* | *- 225 bp* |
| **CBX7** | *yGGGTGGATGGATAAATGGAA* | *CAAAACCRAAATCTCCCTAATCC* | *TGCGTCGCGGAGAA* | *- 519 bp* |

**Supplemental Table 2. Clinicopathologic Parameters of Patients with Breast Cancer in the Test and Validation Sets**

| **Factors** | **Test set (n=108) %** | | | | **Validation set (n=194) %** | | ***P* value** |
| --- | --- | --- | --- | --- | --- | --- | --- |
| **Patient characteristics** |  | | |  |  |  | ***P>0.05*** |
| Age at diagnosis | 48.82±8.78 | | |  | 49.13±8.41 |  |  |
| Age at menarche | 12.84±1.40 | | |  | 12.76±1.10 |  |  |
| BMI at diagnosis(kg/m2) | 24.35±3.08 | | |  | 24.31±3.40 |  |  |
| ***Frequencies*** |  | | |  |  |  | ***P=0.311*** |
| Family history of BC or OC | 14 | | | *12.96* | 27 | *13.92* |  |
| Family history of other tumors | 12 | | | *11.11* | 25 | *12.89* |  |
| History of benign mass | 36 | | | *33.33* | 58 | *29.90* |  |
| **Tumor characteristics** |  | | |  |  |  |  |
| **Size** |  | | |  |  |  | ***P=0.382*** |
| ＜2 cm | 75 | | | *69.44* | 130 | *67.01* |  |
| ≥2 cm | 33 | | | *30.56* | 64 | *32.99* |  |
| **Grade** |  | | |  |  |  | ***P=0.419*** |
| Well differentiated | 24 | | | *22.2* | 37 | *19.1* |  |
| Moderately differentiated | 55 | | | *50.9* | 114 | *58.8* |  |
| Poorly differentiated | 29 | | | *26.9* | 43 | *22.2* |  |
| **Nodal involvement** |  | | |  |  |  | ***P=0.374*** |
| No nodes | 73 | | | *67.6* | 128 | 66.0 |  |
| 1–3 | 31 | | | *28.7* | 51 | 26.3 |  |
| ≥4 | 4 | | | *3.7* | 15 | 7.7 |  |
| **Subtypes** |  | | |  |  |  | ***P=0.19*** |
| Luminal-A | | 75 | *69.4* | | 123 | 63.4 |  |
| Luminal-B | | 13 | *12.0* | | 15 | 7.7 |  |
| Her-2 | | 12 | 11.1 | | 35 | 18.1 |  |
| Triple-negative | | 8 | 7.4 | | 21 | 10.8 |  |
| **Pathological types** | |  |  | |  |  | ***P=0.806*** |
| DCIS | | 35 | 32.4 | | 65 | 33.5 |  |
| IDC | | 34 | 31.5 | | 66 | 34.0 |  |
| IDC_L | | 39 | 36.1 | | 63 | 32.5 |  |

**BMI**, body mass index; **BC**, breast cancer; **OC**, ovary cancer

**Supplement Table 3**. Clinical Pathological Characteristics of Benign and Normal Control Cases.

|  |  |  |  |
| --- | --- | --- | --- |
|  |  | Benign Control Cases (n=300) | Normal Control Cases (n=300) |
| **Age** |  | 49.86±10.30 | 50.44±8.66 |
| **Types of Dieases** |  |  |  |
|  | **Fibroadenoma** | 139 | - |
|  | **Benign Phyllodes Tumors** | 16 | - |
|  | **Mastopathy** | 35 | - |
|  | **Papilloma** | 97 | - |
|  | **Duct Ectasia and Hamartoma** | 13 | - |
|  |  |  |  |

**Supplemental Table 4.** **Methylated Frequencies of Nine Genes in Breast Cancer Tissues from the Test and Validation Sets**

| **Gene Methylated** | **Test set** | | **%** | **Validation set** | **%** | ***P* value** |
| --- | --- | --- | --- | --- | --- | --- |
|  | **(108 cases)** | |  | **(194 cases)** |  |  |
| ***SFN*** | + | 30 | ***27.78*** | 61 | ***31.44*** | ***0.5058*** |
|  | - | 78 |  | 133 |  |  |
|  |  |  |  |  |  |  |
| ***HOXA11*** | + | 41 | ***37.96*** | 88 | ***45.36*** | ***0.2129*** |
|  | - | 67 |  | 106 |  |  |
|  |  |  |  |  |  |  |
| ***P16*** | + | 45 | ***41.67*** | 86 | ***44.33*** | ***0.6544*** |
|  | - | 63 |  | 108 |  |  |
|  |  |  |  |  |  |  |
| ***RASSF1A*** | + | 61 | ***56.48*** | 117 | ***60.31*** | ***0.5169*** |
|  | - | 47 |  | 77 |  |  |
|  |  |  |  |  |  |  |
| ***PCDHGB7*** | + | 82 | ***75.93*** | 156 | ***80.41*** | ***0.3605*** |
|  | - | 26 |  | 38 |  |  |
|  |  |  |  |  |  |  |
| ***hMLH1*** | + | 33 | ***30.56*** | 69 | ***35.57*** | ***0.3775*** |
|  | - | 75 |  | 125 |  |  |
|  |  |  |  |  |  |  |
| ***Wnt5a*** | + | 29 | ***26.85*** | 57 | ***29.38*** | ***0.6406*** |
|  | - | 79 |  | 137 |  |  |
|  |  |  |  |  |  |  |
| ***HOXD13*** | + | 53 | ***49.07*** | 90 | ***46.39*** | ***0.6545*** |
|  | - | 55 |  | 104 |  |  |
|  |  |  |  |  |  |  |
| ***RARβ*** | + | 42 | ***38.89*** | 71 | ***36.6*** | ***0.6933*** |
|  | - | 66 |  | 123 |  |  |

**Supplemental Table 5. Methylation Frequencies for the Nine Genes in Breast Cancer Patients**

|  |  |  |
| --- | --- | --- |
| **Methylated Gene** | **Breast Cancer Tissue (*302 cases*)** | |
|  | Methyl-frequency (%) | |
| *SFN* | *30.13* | |
| ***HOXA11*** | **42.72** | |
| ***P16*** | **43.38** | |
| ***RASSF1a*** | **58.94** | |
| ***PCDHGB7*** | **78.81** | |
| *hMLH1* | *33.77* | |
| *Wnt5a* | *28.48* | |
| ***HOXD13*** | **47.35** | |
| *RARβ* | *37.42* | |
|  |  |  |

**Supplemental Table 6. The List of Co-existing Methylated Genes in Specimens with Different Histopathological Types in Breast Cancer**

| **Histopathological Types** | | **Methylated Gene Amount** | | |
| --- | --- | --- | --- | --- |
|  |  | **1-3 (%)** | **4-6 (%)** | **7-9 (%)** |
|  | *DCIS (cases)* | *24/100 (24%)* | *71/100 (71%)* | *5/100 (5%)* |
|  | *IDC (cases)* | *28/100 (28%)* | *60/100(60%)* | *12/100(12%)* |
|  | *IDC-L (cases)* | *26/102(25.5%)* | *55/102 (53.9%)* | *21/102(20.6%)* |

**Supplemental figures:**

**Supplemental figure 1:**


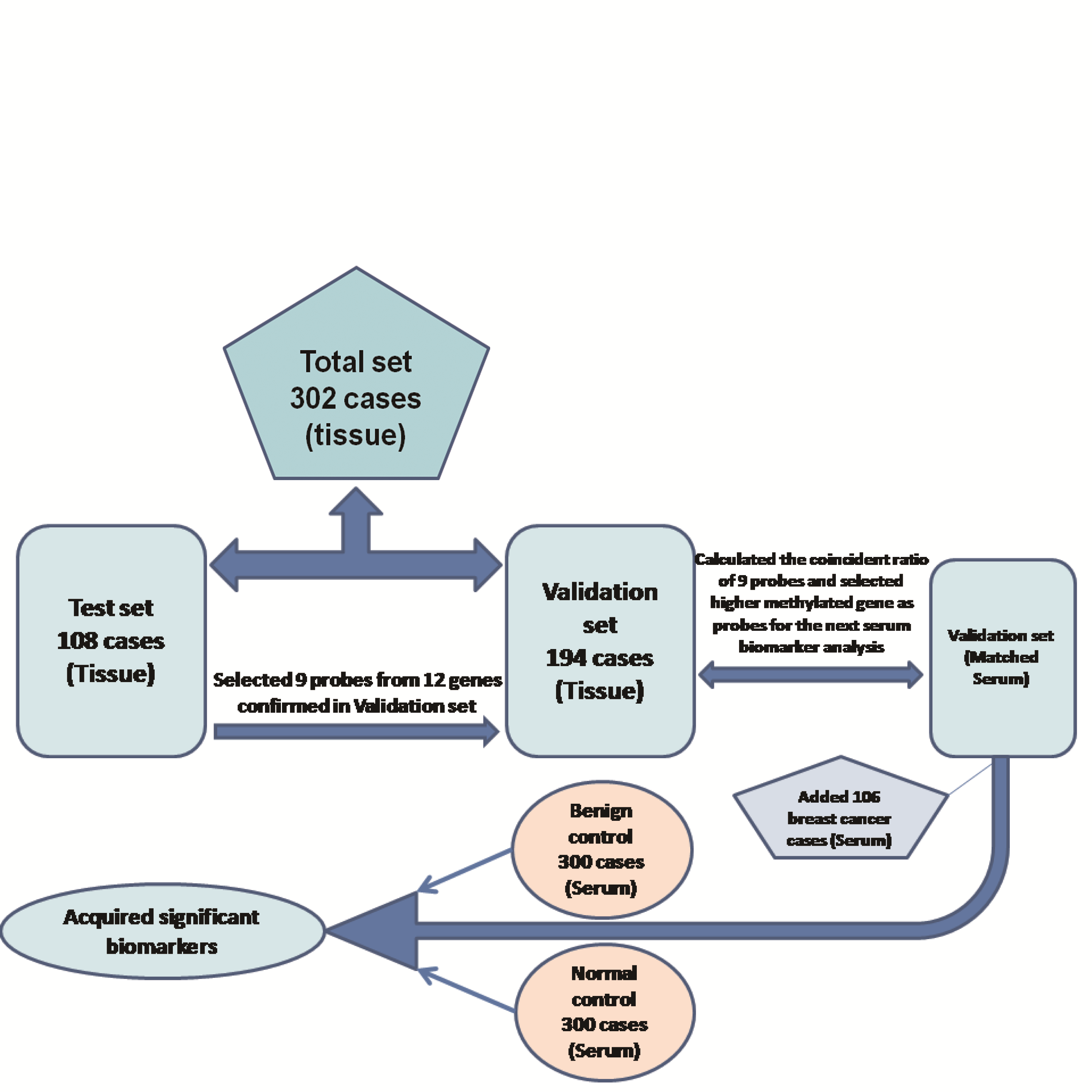


**Supplemental Figure 1:** Overview of the Analyzed Procedure

**Supplemental figure 2:**


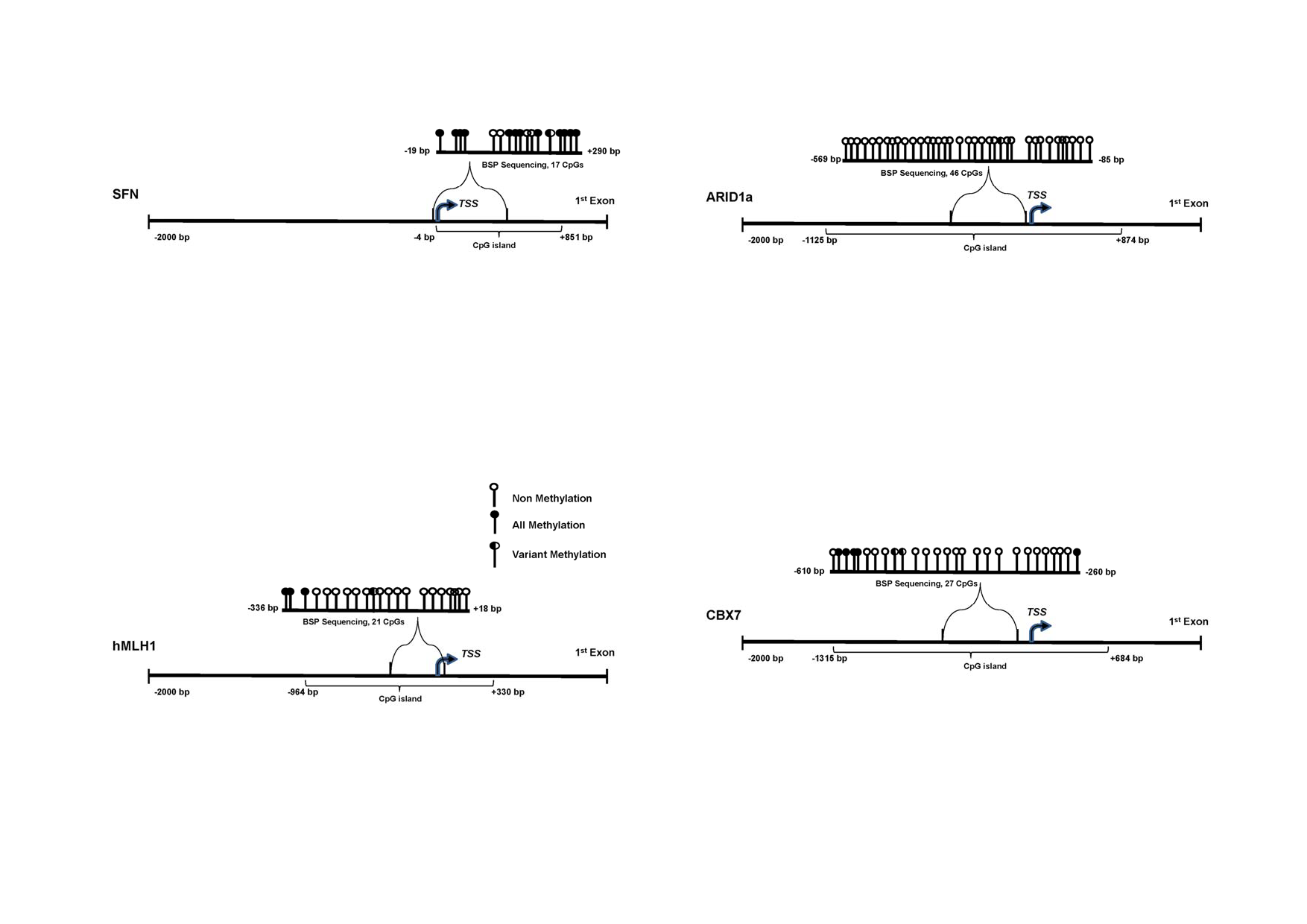


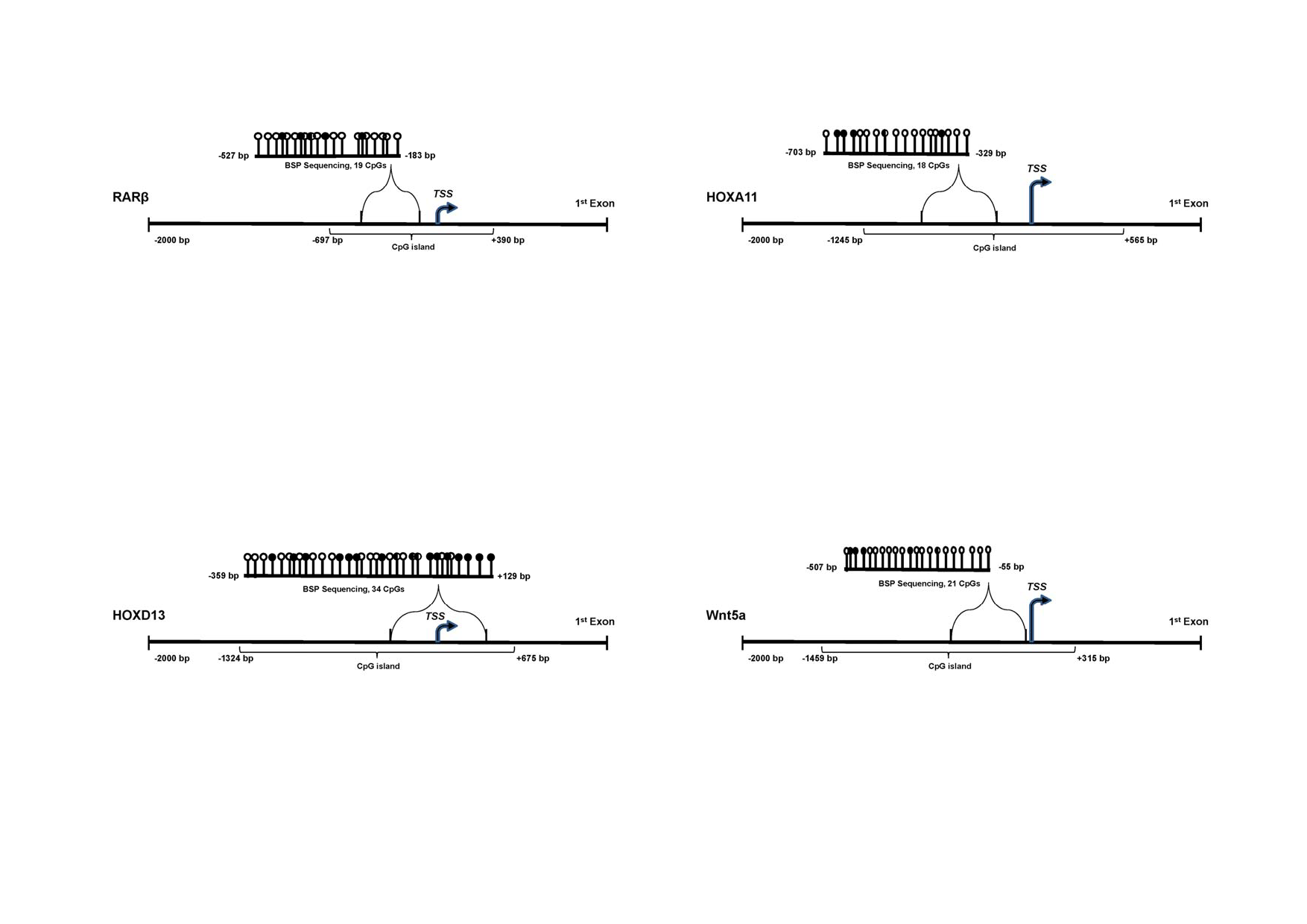


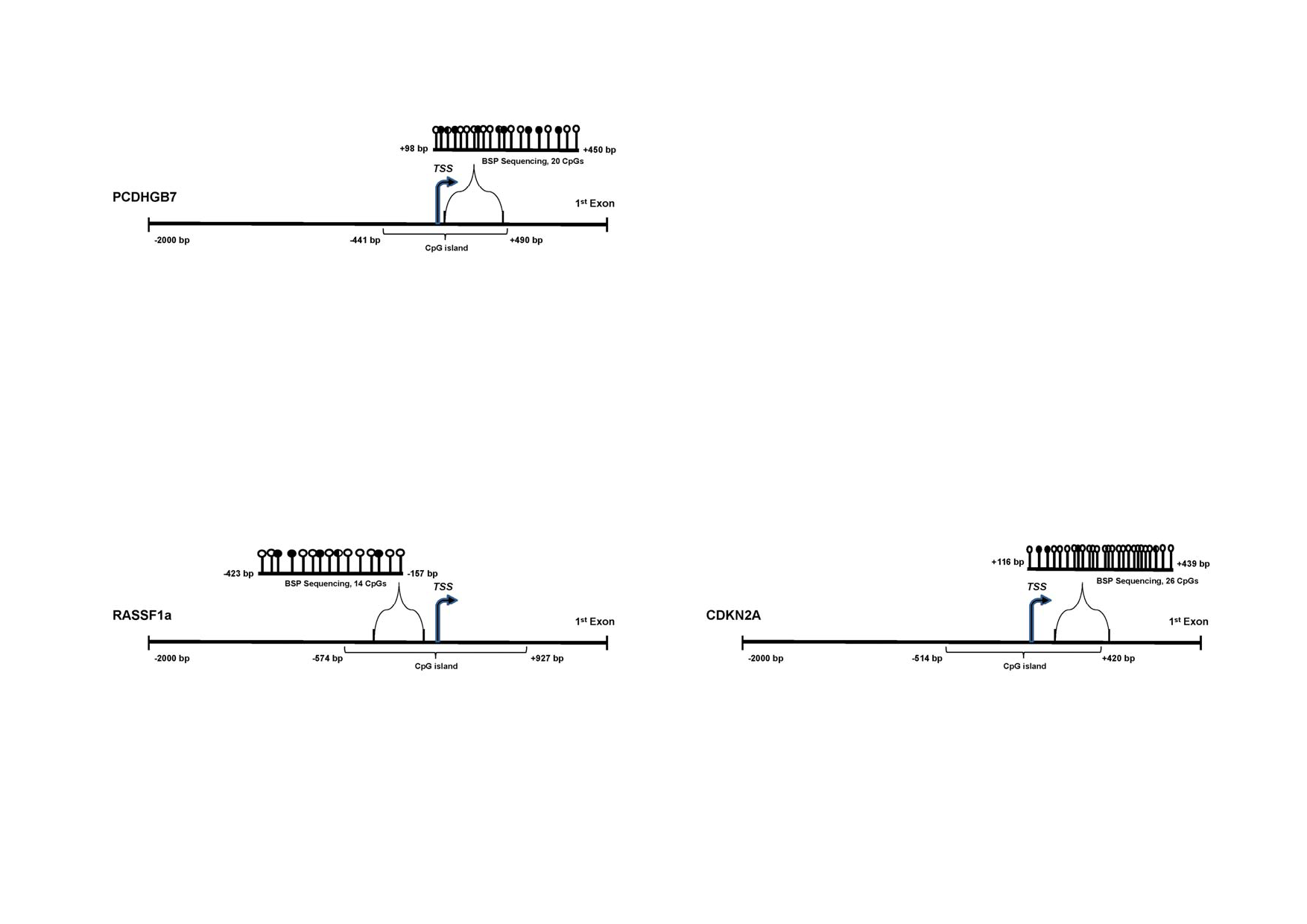


**Supplemental Figure 2:**The BSP Analysis and the Methylated Sites Chosen for All of the Genes

**Supplemental Figure 3:**

**
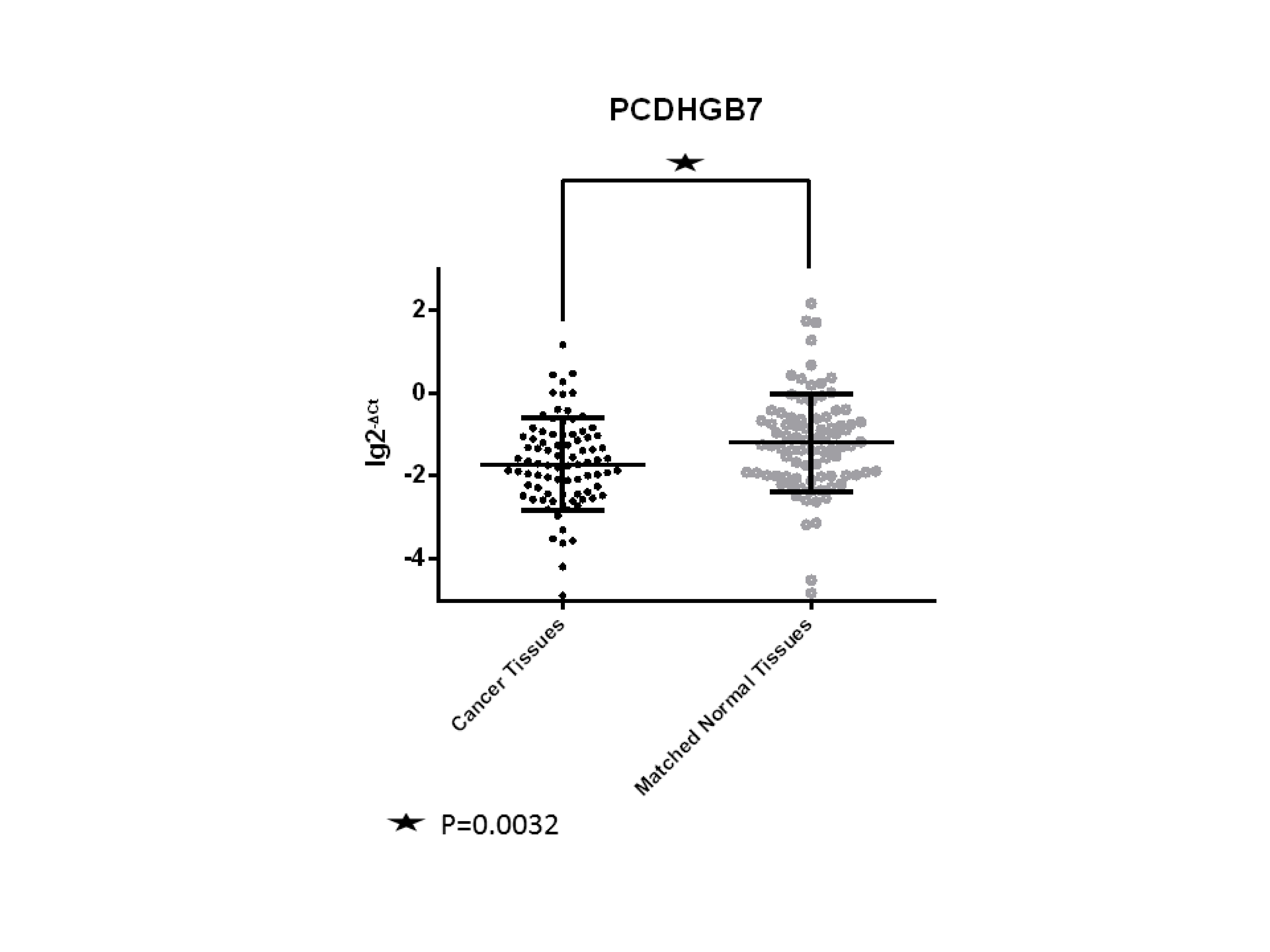
**

**Supplemental Figure 3:** Differential Expression of PCDHGB7 between BC Tissues and Matched-normal Breast Tissues
